# Visualizing the Real Time Reduction of Pulled Elbow Using Point of Care Ultrasound (POCUS)

**DOI:** 10.24908/pocus.v9i2.17961

**Published:** 2024-11-15

**Authors:** David J Mccreary, Mbbs Mrcph, Pgc Us

**Affiliations:** 1 Paediatric Emergency Department, Sunderland Royal Hospital Sunderland UK

**Keywords:** MSK POCUS, Pulled Elbow, Nursemaid's Elbow, Hook, Synovial Fringe

## Abstract

POCUS is a useful tool for correctly identifying pulled elbow. We believe that clinicians working in Pediatric Emergency Departments should be encouraged to embrace using it in cases which are less straightforward – either due to an atypical history or based on examination findings. This will serve to not only increase safety and improve the patient journey, but also to improve the clinician’s confidence in their practice. This is the first ever documented instance where the reduction of pulled elbow has been demonstrated in real time using POCUS.

## Background

Radial head subluxation, known as “pulled elbow” or “nursemaid’s elbow,” is a common presentation to the Paediatric Emergency Department (PED) in children below the age of 5 years. It occurs following longitudinal traction to the child’s forearm (i.e. when their hand or wrist is pulled upward by an adult). The child typically appears comfortable at rest but is unwilling to fully use the affected arm 1. Correct diagnosis is dependent on both a clear history from caregivers along with recognizing the above presentation. It is confirmed by the child’s immediate improvement following manipulative reduction, heralded by the satisfying “click” that is heard and felt when performed by the clinician. Frequently, however, the patient presentation is less straightforward as 50% of children present with an unwitnessed injury or an atypical history 2. In addition, examination can be difficult in a child that is upset or frightened, meaning the precise location of the injury is not always easily identifiable. As a result, many children unnecessarily receive upper limb x-rays 3, and others undergo attempted reduction for suspected pulled elbow when a fracture is the cause of their pain.

In our PED, point of care ultrasound (POCUS) for upper limb injuries is undertaken by those who have received training and who have demonstrated sufficient POCUS competence. POCUS has been shown to be a highly effective tool in our department for patients presenting with undifferentiated elbow injuries, particularly when the history is incomplete or lacking. In other studies, POCUS has been demonstrated to have both a high sensitivity and specificity for correctly identifying pulled elbow 4, 5, 6, 7. The positive sonographic features of the “hook sign” – where the supinator muscle overlying the radiocapitellar joint extends farther than expected into the joint space, and has a hooked appearance along with an increased prominence of the synovial fringe which appears to fall into the joint space – have been the focus of much recent literature 5, 8, 9, 10. 

## Case Presentation

An 18-month-old girl presented to the PED after her mother lifted her up from the floor while holding her wrists. She cried immediately and appeared to be refusing to use the right arm thereafter. On examination, she appeared comfortable at rest, with her right arm held adducted and in 10° of flexion at the elbow joint. She refused to use the right arm despite encouragement with toys and stickers. POCUS was undertaken, and did not reveal elbow joint effusion when viewed in the transverse and longitudinal planes at the olecranon fossa. This excluded the possibility of an intracapsular bony injury such as a supracondylar or radial head fracture. Views were obtained along the radiocapitellar joint for suspected pulled elbow, and sonographic signs for this were present: a positive hook sign with a prominent synovial fringe confirmed the diagnosis. 

In this video we demonstrate a real time reduction of a pulled elbow under POCUS visualization (four separate video clips have been amalgamated into one to make it easier to appreciate).

At the beginning of the video (S1), the typical appearances of pulled elbow are evident with the supinator muscle manifesting the hook sign as extending proximally into the joint space and with a prominent synovial fringe with its pointed appearance. The video is labelled in order to show the most proximal aspect of the supinator muscle (arrow). Note how this muscle begins to retract distally when the manipulation commences, in this case carried out by the hyperpronation method 11. As the pronation continues, the muscle returns to its expected position. This signifies that the reduction has been successful. Following the reduction, the synovial fringe appears much less prominent and takes on a normal appearance. 

## Discussion

Tsai et al. describe the use of “dynamic” ultrasound examination as an important diagnostic and therapeutic evaluation tool for the treatment of a nursemaid’s elbow in 13 patients, focussing mainly on the appearance of the partial “ellipse sign” seen in transverse views of the radial head as a result of the posterior synovial fringe becoming entrapped between the radial head and annular ligament 12. There have been no further studies evaluating this sign. We too recommend serial ultrasound scans pre- and post-reduction in order to confirm diagnosis and proper reduction. However, this is the first documented example where reduction is demonstrated in real time using POCUS.

## Conclusion

POCUS is a useful tool for correctly identifying pulled elbow. We believe that clinicians working in PEDs should be encouraged to embrace using it in cases which are less straightforward – either due to an atypical history or based on examination findings. This will serve to not only increase safety and improve the patient journey, but also to improve the clinician’s confidence in their practice. This is the first documented instance where the reduction of pulled elbow has been demonstrated in real time using POCUS.

## Informed Consent Statement 

Consent was fully informed at the time of undertaking the manipulation and obtained verbally. All patient identifiable data has been removed from any included scans. 

## Disclosure Statement 

The authors declare that they have no competing interests.

## Supplementary Material

• Video S1
